# A Reaction–Diffusion Model for Capturing Mass Loss and Microstructure Evolution in Enzymatic Degradation of Poly(*ε*-Caprolactone) Films

**DOI:** 10.3390/polym18101248

**Published:** 2026-05-20

**Authors:** Nanshin Nansak, Leo Creedon, Denis O’Mahoney, Ramen Ghosh, Marion McAfee

**Affiliations:** 1 Centre for Mathematical Modelling and Intelligent Systems for Health and Environment, Atlantic Technological University, F91 YW50 Sligo, Ireland; nanshin.nansak@research.atu.ie (N.N.); leo.creedon@atu.ie (L.C.); denis.omahoney@atu.ie (D.O.); ramen.ghosh@atu.ie (R.G.); 2 Department of Mechanical Engineering, Atlantic Technological University, H91 T8NW Galway, Ireland

**Keywords:** enzymatic degradation, reaction–diffusion model, semicrystalline polymers, poly(ε-caprolactone), crystallinity, Bayesian inference, Sobol sensitivity analysis, lipase, microstructure

## Abstract

The microstructure of semicrystalline bioresorbable polymers is central to their biomedical performance because the crystalline content influences both the mechanical stability and the degradation behaviour. Experimental studies have shown that crystallinity evolves concurrently with mass loss during enzymatic degradation. However, most existing models represent the material as a single homogeneous structure, preventing them from capturing this microstructural evolution or the state-selective mechanisms that drive it. We present a one-dimensional partial differential equation model for the enzymatic degradation of thin films, which treats the crystalline and amorphous states as distinct reactive components. Calibrated to poly(ε-caprolactone) (PCL) degraded by *Candida antarctica* lipase in vitro, the model accurately reproduces both the observed weight-loss profile and the concurrent decline in crystallinity. Parameter uncertainty analysis indicates that while there are varying degrees of confidence in individual parameter values, the overall model predictive uncertainty is well constrained. Parameter sensitivity analysis shows that the amorphous catalytic rate (the rate at which the enzyme degrades the amorphous region) is the dominant driver of degradation dynamics. The identified model parameters are used to explore the role of film thickness on the rates of mass and crystallinity loss. It was found that thin films remain largely reaction-limited, whereas thicker specimens become increasingly transport-influenced, with slower degradation and delayed structural evolution in the material interior. The model provides a useful tool to explore the effect of changing PCL film thickness on degradation rate and crystallinity-related properties without extensive experimentation.

## 1. Introduction

Semicrystalline polymers such as poly-ε-caprolactone (PCL) are extensively employed in the fabrication of bioresorbable films for medical applications. These films are effective as surgical aids, including resorbable hernia meshes designed to provide a bioactive environment for cell development and barrier membranes used in laparoscopy to prevent tissue adhesion [1,2,3,4]. In tissue engineering, PCL films function as specialised patches for cartilage, skin, and bone–tendon interface repair, as well as protective coatings for dental implants to enhance cell–material interactions. Their clinical success is underpinned by FDA approval and the ability to undergo slow, controlled degradation via hydrolysis or enzymolysis into non-toxic byproducts such as 6-hydroxycaproic acid [3]. The rubbery state of PCL at body temperature, combined with the tunable transparency, permeability, and mechanical flexibility afforded by PCL, makes these films ideal for sustained structural support and long-term drug delivery. Such characteristics have further enabled “smart” film-based systems, including shape-memory foams for aneurysm treatment and enzyme-responsive therapeutic platforms [5,6]. A diverse range of enzymes mediate the enzymatic degradation of bioresorbable polymers [7]. Upon implantation, these materials typically elicit a wound healing response, initiating the recruitment of inflammatory cells and activation of the immune system [8]. These cellular interactions promote the secretion of degradative enzymes, particularly by macrophages and multinucleated giant cells [4]. In vivo evidence of enzymatic degradation has been found in animal studies; for example, Yang et al. [9] reported substantial subcutaneous erosion of poly(trimethylene carbonate) (PTMC) rods in rats due to lipase activity, in contrast to negligible degradation under purely hydrolytic conditions. Furthermore, this enzymatic process is actively leveraged in the development of enzyme-responsive polymeric systems capable of releasing therapeutic agents selectively in pathological environments, such as cancerous tumours with elevated enzyme concentrations [5,6].

PCL exhibits more complex enzymatic degradation than amorphous polymers because crystalline lamellae and amorphous domains differ fundamentally in accessibility and reactivity. Accordingly, extensive studies of PCL consistently show that degradation is governed by interacting factors, including enzyme type and origin, initial crystallinity and morphology, and surface accessibility. A recurring observation is that crystallinity evolves during degradation, which may be due to preferential or non-preferential enzymolysis of the polymer states. In systems where the amorphous regions are degraded more rapidly than the crystalline regions, the crystalline fraction of the specimen may increase because the loss of amorphous material leaves a residue which is mainly crystalline [10,11,12,13,14]. This behaviour has been observed across a range of bacterial lipases and cutinases, both in surface-erosion systems and in polymer specimens with enzyme embedded in the polymer matrix [9,10,15]. Conversely, other studies report a net decrease in crystallinity, arguing that enzymolysis is not confined to amorphous domains and that crystalline regions are also substantially attacked [16,17,18]. This has been linked in particular to certain fungal lipases, where random chain scission is thought to disrupt lamellar order [16,19], and further supported by evidence that accessibility of enzyme to the surface and the role of porosity in accessibility to the bulk of the polymeric device can be more significant factors in the degradation rate than the percentage crystallinity of the device [9]. Degradation kinetics reflect enzyme-specific behaviour: cutinases and lipases have been shown to produce markedly different mass-loss profiles and surface morphologies on identical PCL substrates [16,20], with cutinases often producing more linear, surface-controlled kinetics and lipases producing multi-stage profiles associated with pore formation and progressive deeper penetration [17,20].

Several kinetic frameworks have been proposed for enzymolysis. Mukai et al. [21] introduced a surface-erosion model for enzymatic degradation of poly[(R)-3-hydroxybutyrate] (P[(R)-3HB]) films, in which the overall rate is governed primarily by enzyme adsorption to the polymer surface. Despite the semicrystalline nature of P[(R)-3HB], however, the formulation treated the reactive surface as homogeneous, without distinguishing amorphous and crystalline regions or accounting for morphology evolution during degradation. Duguay et al. [22] developed a detailed mechanistic framework for in vitro cholesterol-esterase degradation of a poly(ester-urea-urethane), integrating surface dynamics, enzyme adsorption, solvolysis, enzymatic bond cleavage, and soluble-product degradation. While comprehensive, the resulting 31-equation system is difficult to calibrate from typical degradation datasets, which generally provide only a limited number of observable endpoints such as mass loss. Timmins and Lenz [23] further demonstrated that experimentally observed rate laws for PHB and PHBV degradation can deviate from classical Michaelis–Menten behaviour, and derived a surface-based model fitted by least-squares analysis, again without resolving the crystalline and amorphous contributions separately. Sridhar and Vernerey [24] presented a one-dimensional centro-symmetric reaction–diffusion model for localised enzymatic degradation of polymers, formulated in terms of enzyme diffusion and reduction in polymer cross-link density. Their main contribution was to characterise the propagation of a fuzzy degradation interface and derive scaling laws for its speed and width from competing transport and degradation timescales. While this provides important physical insight into reaction–transport coupling, the model treats the polymer as a homogeneous network and does not resolve semicrystalline microstructure, state-selective enzymolysis, crystallinity evolution, or simultaneous fitting to crystallinity and mass-loss data. More recently, Nansak et al. [25] proposed a mechanistic model for PLA enzymolysis incorporating product inhibition, where the accumulation of acidic degradation products slows or even stops further enzymatic degradation; however, this work considered amorphous PLA and did not extend to semicrystalline systems where crystallinity evolves concurrently with degradation.

For semicrystalline substrates, mass loss alone cannot distinguish preferential amorphous attack from amorphisation-driven crystallinity decrease. The same weight-loss trajectory can arise from preferential attack of amorphous domains (which would increase crystallinity) or from non-preferential enzymatic attack (which would decrease it). Crystallinity measured concurrently with mass loss therefore provides independent information on the relative decay of the two states, improving the identifiability of state-specific kinetic parameters. Beyond identifiability, crystallinity carries direct design relevance: mechanical integrity, drug-release kinetics, and resorption timescale are all sensitive functions of the crystalline fraction, so predictive design requires a model that captures microstructural evolution, not just mass loss.

While existing enzymatic degradation models can reproduce mass-loss trends, they typically treat semicrystalline polymers as homogeneous substrates, thereby collapsing crystalline and amorphous domains into a single reactive state [21,23,25]. Consequently, they do not explicitly capture state-selective enzymolysis, the dynamic evolution of crystallinity, or the coupled feedback between porosity development and transport-limited enzyme diffusion. A mechanistic framework that links these interacting processes remains largely absent from the literature.

The present work develops a reaction–diffusion model in which crystalline and amorphous regions undergo distinct enzyme-binding and enzymolysis reactions, enzyme mobility is governed by porosity-dependent diffusivity, and crystallinity is not treated as a constant but evolves dynamically as a model variable. This formulation enables simultaneous prediction of mass loss and crystallinity trajectories.

Our main contributions are:

- A two-state reaction–diffusion PDE model suitable for modelling thin films of bioresorbable semicrystalline polymers. The model captures the different rates of enzyme attack on crystalline and amorphous regions which have been observed in vitro.
- An explicit amorphisation pathway that captures the effect observed in certain polymer–enzyme systems, where the enzyme disrupts the order of crystalline regions prior to degradation.
- A porosity-dependent effective diffusivity function coupling microstructural evolution to enzyme transport.
- Simultaneous calibration to weight-loss and crystallinity data, improving identification of state-specific kinetic parameters.
- Global, time-dependent parameter sensitivity analysis quantifying which kinetic steps govern the model dynamics and when.
- The model is exploited to allow for the prediction of mass and crystallinity loss in varying thicknesses of films, based on the parameters identified from a single experiment. Although the effective diffusion constant is uncertain, the model clearly captures the transition from thin films, which are dominated by the enzymatic degradation reaction (and show no difference in mass or crystallinity loss rates below a certain threshold thickness), and thicker films where transport of the enzyme into the bulk plays a greater role, resulting in slower mass and crystallinity loss.

The remainder of the manuscript is organised as follows: Section 2 presents the model development, while Section 3 reports a case study of the fitting of the enzymatic degradation model to an in vitro study of PCL degraded by lipase. Section 4 outlines the model sensitivity analysis, Section 5 discusses the findings, and Section 6 concludes the paper.

## 2. Model Development

### 2.1. Degradation Kinetics

The enzymatic degradation of bioresorbable semicrystalline polymers proceeds through physicochemically governed processes involving enzyme transport, adsorption, and enzyme-catalysed chain scission. When a polymeric device is immersed in an aqueous medium containing enzymes, enzyme molecules diffuse through the solution and adsorb onto accessible polymer surfaces [26,27]. Following adsorption, enzymatic hydrolysis of polymer chains initiates at the polymer–enzyme interface via random or end-chain scission, generating low-molecular-weight oligomers and monomers that are released as soluble degradation products. In the case of PCL, the primary degradation product is 6-hydroxycaproic acid [16,19].

As scission proceeds, polymer microstructure may evolve via amorphisation (disruption of crystalline domains) or recrystallisation (reordering of scission-shortened chains), depending on enzyme type, polymer chemistry, and processing history [9,16]. For semicrystalline polymers such as PCL, this evolution is governed by state-selective enzymolysis: amorphous regions, with lower packing density and higher chain mobility, are typically hydrolysed more readily than crystalline lamellae [28,29]. Accordingly, crystallinity may increase or decrease depending on the relative degradation rates of amorphous and crystalline states, the transition rate from crystalline to amorphous and the enzyme’s mode of action [16,19]. Lipases are often reported to induce substantial structural disruption via random chain scission that progressively affects both amorphous and crystalline domains, whereas cutinases tend to operate by surface-controlled erosion with limited disruption to the crystalline structure [20]. These mechanistic differences also govern macroscopic degradation kinetics: surface-eroding enzymes typically produce near-linear mass loss, while penetrative enzymes can generate multi-stage profiles with an initially rapid state followed by a slower state as enzyme activity diminishes and accessible substrate becomes depleted.

**Figure 1:**
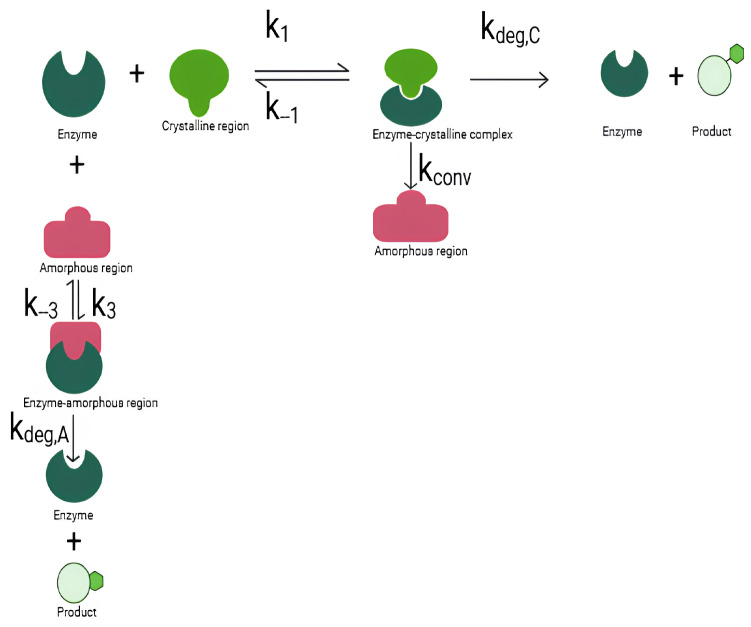
A schematic of the reaction.

Shi et al. [16] showed that lipase degrades PCL by a more penetrative and disruptive mechanism than a surface-limited one, producing substantial weight loss and major structural changes in the polymer, such as the formation of surface holes, rounded pores around cracks, and eventually a large cavity, with some pores extending through the film. It was also found that lipase does not act only on amorphous domains but also disrupts crystalline regions, as evidenced by the marked drop in crystallinity. A reaction scheme representing the enzymatic degradation of PCL by Lipase studied in Shi et al. [16] is shown in Figure 1 and Equation (1).

(1)
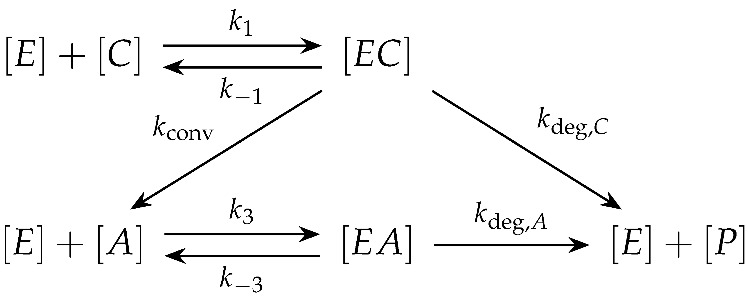


Here, [E], [C], [A], [EC], [EA], and [P] denote the concentrations of free enzyme, crystalline polymer, amorphous polymer, enzyme–crystalline complex, enzyme–amorphous complex, and soluble degradation product, respectively. The rate constants $k_{1}$ and $k_{3}$ govern forward binding of the enzyme to the crystalline and amorphous states, respectively; $k_{-1}$ and $k_{-3}$ are the corresponding dissociation rates; $k_{\mathrm{conv}}$ is the rate of conversion of crystalline material to amorphous material upon enzyme binding; and $k_{\deg,C}$ and $k_{\deg,A}$ are the catalytic enzymolysis rates of the crystalline and amorphous complexes, respectively. The $k_{\mathrm{conv}}$ pathway reflects the experimentally observed partial disordering of crystalline domains before complete enzymolysis.

Equations (2)–(7) are obtained by combining the mass-action kinetics of the reaction scheme in Equation (1) with Fick’s second law applied to the free enzyme [E]. For each species *X*, a pointwise mass balance of the form $\partial \left[X\right]/\partial t=\nabla \cdot \left(D_{X}\nabla \left[X\right]\right)+R_{X}$ is written, where $R_{X}$ is the net reaction rate obtained from the law of mass action and $D_{X}$ is the species diffusivity. The diffusion term in Equation (2) represents bulk diffusion of the enzyme through the evolving porous structure of the film, rather than surface diffusion. As degradation proceeds, polymer is converted to soluble products and removed from the matrix, increasing local porosity and hence the effective diffusivity $D_{e}\left(\phi \right)$. The resulting reaction–diffusion system is:

(2) $$\begin{array}{ccc} \frac{\partial [E]}{\partial t} & = & \frac{\partial }{\partial x}\;\left(D_{e}\left(\phi \right)\;\frac{\partial [E]}{\partial x}\right)-k_{1}\left[E\right]\left[C\right]+k_{-1}\left[EC\right]-k_{3}\left[E\right]\left[A\right]+k_{-3}\left[EA\right]\\ & & +k_{\mathrm{conv}}\left[EC\right]+k_{\deg,C}\left[EC\right]+k_{\deg,A}\left[EA\right], \end{array}$$

(3) $$\frac{\partial [C]}{\partial t}=-k_{1}\left[E\right]\left[C\right]+k_{-1}\left[EC\right],$$

(4) $$\frac{\partial [A]}{\partial t}=-k_{3}\left[E\right]\left[A\right]+k_{-3}\left[EA\right]+k_{\mathrm{conv}}\left[EC\right],$$

(5) $$\frac{\partial [EC]}{\partial t}=k_{1}\left[E\right]\left[C\right]-k_{-1}\left[EC\right]-k_{\mathrm{conv}}\left[EC\right]-k_{\deg,C}\left[EC\right],$$

(6) $$\frac{\partial [EA]}{\partial t}=k_{3}\left[E\right]\left[A\right]-k_{-3}\left[EA\right]-k_{\deg,A}\left[EA\right],$$

(7) $$\frac{\partial [P]}{\partial t}=k_{\deg,C}\left[EC\right]+k_{\deg,A}\left[EA\right].$$

Here, *x* denotes the spatial coordinate in the through-thickness direction, and *t* denotes time. The crystalline and amorphous state equations contain no diffusion terms because the polymer phases are treated as immobile within the continuum formulation, and degradation progresses into the bulk only as the enzyme penetrates the material. Equation (7) accounts for the formation of soluble degradation products from polymer breakdown, with [P] acting as the terminal mass sink of the reaction network. The product equation similarly contains no diffusion term; soluble degradation products are assumed to be rapidly removed by the well-mixed enzyme bath, consistent with the in vitro conditions of [16], so their accumulation within the matrix is negligible.

### 2.2. Spatial Domain and Boundary Conditions

**Figure 2:**
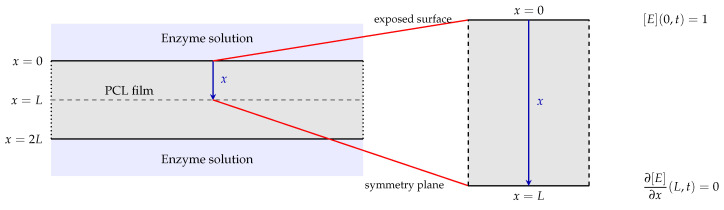
PCL film cross-section and one-dimensional half-thickness domain (0≤x≤L).

In this work we focus on modelling the enzymatic degradation of film (Figure 2) where the thickness is significantly less than the width and length. Because of this, enzyme transport is approximated as one-dimensional in the through-thickness coordinate *x*. Since the films were fully immersed in the enzyme buffer, both faces were exposed equally, so the concentration profile is symmetric about the axis. It therefore suffices to model half the thickness, x∈[0,L], using a Dirichlet condition at the exposed surface and a no-flux condition at the symmetry plane (Figure 2).

The initial conditions for 0<x≤L are

(8) $$\begin{array}{cc} \left[E](x,0\right) & =0,\;\left[C\right]\left(x,0\right)=C_{0},\;\left[A\right]\left(x,0\right)=A_{0},\\ \left[EC](x,0\right) & =0,\;[EA](x,0)=0,\;[P](x,0)=0, \end{array}$$

where $C_{0}$ and $A_{0}$ are the initial crystalline and amorphous polymer fractions, respectively. The polymer variables [C] and [A] are expressed as dimensionless volume fractions, normalised by the initial total polymer volume, so that $\left[C\right]\left(x,0\right)+\left[A\right]\left(x,0\right)=C_{0}+A_{0}=1$, with $C_{0}=0.302$ in our case study. The free enzyme concentration [E] is normalised by the bulk bath concentration, so that [E]∈[0,1]. The crystallinity $\chi_{c}\left(t\right)$ corresponds to the fraction of crystalline polymer relative to the remaining polymer content and is computed from the model as $\chi_{c}\left(t\right)=\frac{\int_{0}^{L}\left[C\right]\left(x,t\right)\;dx}{\int_{0}^{L}\left(\left[C\right]\left(x,t\right)+\left[A\right]\left(x,t\right)\right)\;dx}$, where only the polymer regions [C] and [A] are included, as enzyme, complexes, and soluble degradation products are removed during washing and drying prior to differential scanning calorimetry (DSC) measurement. Since all bracketed quantities are dimensionless, the rate constants $k_{1}$, $k_{-1}$, $k_{3}$, $k_{-3}$, $k_{\mathrm{conv}}$, $k_{\deg,C}$, and $k_{\deg,A}$ carry units of $h^{-1}$. At the exposed surface, the normalised enzyme concentration is fixed at the bath value,

(9) [E](0,t)=1,t>0,

and at axis a no-flux condition is imposed,

(10) $${\left.\frac{\partial [E]}{\partial x}\right|}_{x=L}=0,\;t>0.$$

### 2.3. Porosity-Dependent Enzyme Diffusivity

Although degradation initiates at the polymer surface, the progressive formation of pores and cracks provides pathways for enzyme diffusion into the bulk [16,30]. Enzyme transport is therefore modelled as diffusion with a porosity-dependent effective diffusivity $D_{e}\left(\phi \left(x,t\right)\right)$, following the approach of [31]. The local porosity ϕ is defined as the volume fraction of the domain not occupied by solid polymer:

(11) ϕ(x,t)=1−([C]+[A]).

The porosity function does not distinguish between crystalline and amorphous contributions. At the start of degradation (ϕ=0) the polymer is dense; as polymer is consumed (ϕ→1) the effective diffusivity increases. The effective diffusivity is given by

(12) $$D_{e}\left(\phi \right)=D_{e0}\left[1+\alpha_{e}\;\phi \right],$$

where $D_{e0}$ [mm h^−1^] is the baseline the enzyme diffusivity in the dense polymer and $\alpha_{e}$ is a dimensionless coupling constant.

This linear-in-porosity function was adopted as a simple monotonic form that reduces to $D_{e0}$ in the dense polymer, increases smoothly as pores form, and introduces only one additional transport parameter. An analogous porosity–diffusivity coupling appears in Wang et al. [31], where a value of 4.5 was used for product diffusion in bulk hydrolytic degradation. In the present enzymatic setting, $\alpha_{e}=1$ was adopted: a preliminary sensitivity check over the range [0.5,5.0] confirmed that neither the weight loss nor the crystallinity predictions are appreciably affected by the choice of $\alpha_{E}$, and unity is the simplest admissible value within this range.

### 2.4. Numerical Implementation

The PDE system (2)–(7) was solved using the method of lines. The spatial domain x∈[0,L], with L=0.25 mm, was discretised on a uniform grid. A grid-convergence study was performed using N=25, 50, and 100 nodes, and no appreciable changes were observed in the numerical solution. Accordingly, N=25 was retained as a computationally efficient choice, giving a grid spacing of Δx=L/(N−1)≈0.0104 mm. The diffusion operator was approximated using a second-order finite-difference scheme with arithmetic-mean interface diffusivities. The Dirichlet condition at x=0 was enforced by fixing the first node, and the Neumann condition at x=L was enforced using a half-control-volume correction. The resulting system of 6×25=150 stiff ODEs was integrated using the backwards-differentiation formula (BDF) method (ode15s in MATLAB R2025a; SciPy version:1.13.1 BDF in the Python fitting code), with relative tolerance $10^{-6}$, absolute tolerance $10^{-9}$, and a maximum step size of 0.5 h.

## 3. Case Study of Enzymatic Degradation Model Fitting

The enzymatic degradation model developed in this study (Equations (2)–(7)) was parameterised using experimental data reported by Shi et al. [16], which characterises the degradation kinetics of poly(ε-caprolactone) (PCL) films under lipase-driven conditions. In their protocol, PCL films (30×10×0.5 mm) were incubated with *Candida antarctica* lipase (45 U/mL) in potassium phosphate buffer (0.1 M, pH 7.2) at 45 °C, and both weight loss and crystallinity were monitored at successive time points over 72 h. This dataset is selected for its rich data on mass loss and crystallinity evolution over the degradation timescale and serves as a model for enzymes which act to disrupt the order of crystalline lamellae, inducing amorphisation. The degradation timescale is accelerated relative to in vivo conditions, which is a common approach for initial studies into anticipated in vivo behaviour [32].

*Candida antarctica* lipase is a well-characterised fungal lipase widely employed in biodegradation studies of aliphatic polyesters [16,17]. It acts via random chain scission of ester bonds and has been shown to degrade both the amorphous and crystalline domains of PCL, making it a suitable model enzyme for studying coupled mass loss and crystallinity evolution [16,19]. Both pH and temperature are held constant throughout the degradation period by the buffered, temperature-controlled setup, so they act as experimental controls rather than dynamic variables in the model. In the dataset of Shi et al. [16], differential scanning calorimetry (DSC) measurements revealed a decrease in crystallinity throughout the degradation period, indicating that enzymatic attack was not confined to the amorphous domains but extended to the crystalline lamellae. Both the weight-loss profile and the crystallinity data were used simultaneously to calibrate the model.

### 3.1. Parameter Estimation

The goal of the optimisation was to estimate the parameter vector

(13) $$\theta =[k_{1},\;k_{-1},\;k_{3},\;k_{-3},\;k_{\mathrm{conv}},\;k_{\deg,C},\;k_{\deg,A},\;D_{e0}]$$

such that the model output simultaneously best fits both the experimental weight-loss measurements $y_{WL}=\left[y_{WL,1},\ldots ,y_{WL,n}\right]$ and the differential scanning calorimetry (DSC) crystallinity measurements $y_{\chi_{c}}=\left[y_{\chi_{c},1},\ldots ,y_{\chi_{c},m}\right]$, where *n* and *m* denote the number of observations in the weight-loss and crystallinity datasets, respectively. The problem was then formulated as a weighted, bound-constrained nonlinear least-squares minimisation:

(14) $$\min_{\theta \in D}\;L\left(\theta \right)=\sum_{i=1}^{n}{\left[\frac{y_{WL}\left(t_{i};\theta \right)-y_{WL,i}}{\sigma_{WL,i}}\right]}^{2}+\sum_{j=1}^{m}{\left[\frac{y_{\chi_{c}}\left(t_{j};\theta \right)-y_{\chi_{c},j}}{\sigma_{\chi_{c},j}}\right]}^{2}$$

where *n* and *m* are the number of weight-loss and crystallinity measurements, respectively, and $y_{WL}\left(t_{i};\theta \right)$ and $y_{\chi_{c}}\left(t_{j};\theta \right)$ are the model-predicted weight loss and crystallinity at times $t_{i}$ and $t_{j}$, obtained by numerical solution of Equations (2)–(7). The quantities $\sigma_{WL,i}$ and $\sigma_{\chi_{c},j}$ denote the corresponding measurement standard deviations, taken from the experimental uncertainties reported by Shi et al. [16]. Dividing each residual by its standard deviation places both datasets on a common dimensionless scale, ensuring that the weight-loss and crystallinity data contribute comparably to the objective. The feasible domain is defined as $D=\prod_{j=1}^{8}\left[\theta_{j}^{\min},\theta_{j}^{\max}\right]$, with individual parameter bounds given by (15), which was obtained by trial and error.

(15) $$\begin{array}{cc} k_{1} & \in \left[10^{-6},50\right],\;k_{-1}\in \left[10^{-6},50\right],\;k_{3}\in \left[10^{-6},50\right],\;k_{-3}\in \left[10^{-6},50\right],\\ k_{\mathrm{conv}} & \in \left[10^{-6},50\right],\;k_{\deg,C}\in \left[10^{-6},50\right],\;k_{\deg,A}\in \left[10^{-6},50\right],\;D_{e0}\in \left[10^{-12},50\right] \end{array}$$

**Figure 3:**
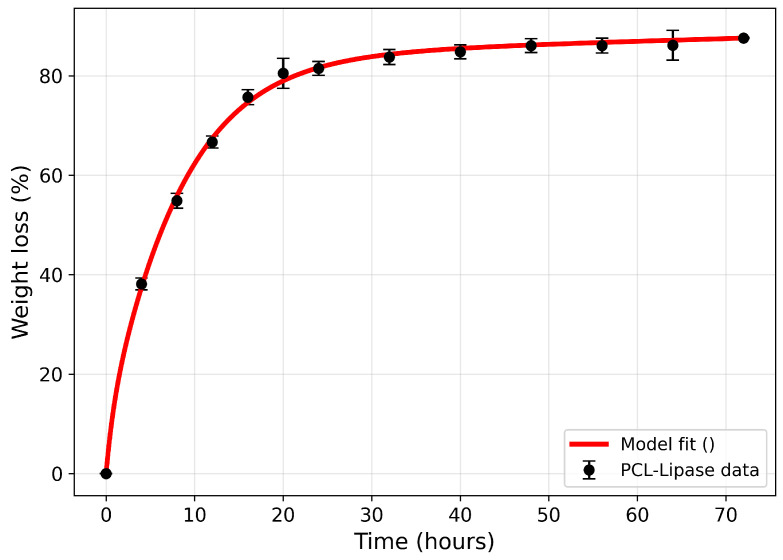
Model fit to PCL–lipase weight loss $R^{2}=0.99$.

**Figure 4:**
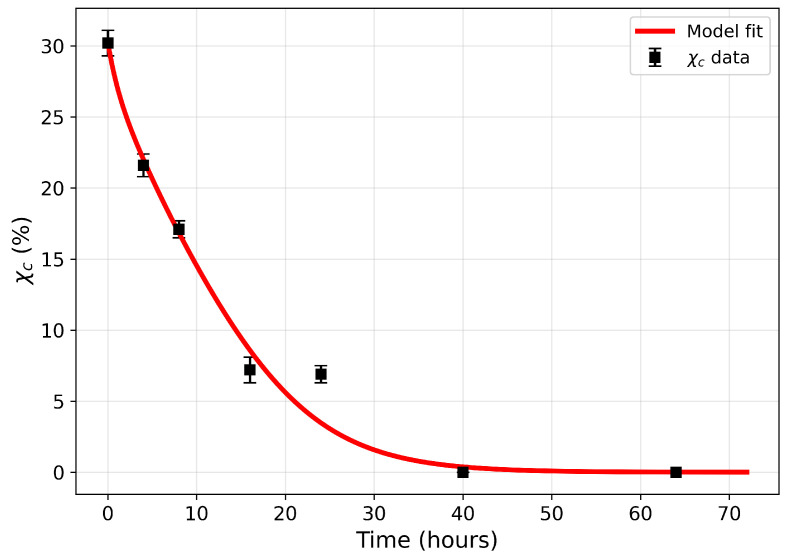
Model fit to PCL–lipase crystallinity ($\chi_{c}$) $R^{2}=0.98$.

The joint fit to the weight-loss and crystallinity datasets is shown in Figure 3 and Figure 4, and the corresponding parameter estimates are reported in Table 1. The model fits the weight-loss data with $R^{2}=0.99$ and the crystallinity data ($\chi_{c}$) with $R^{2}=0.98$.

**Figure 5:**
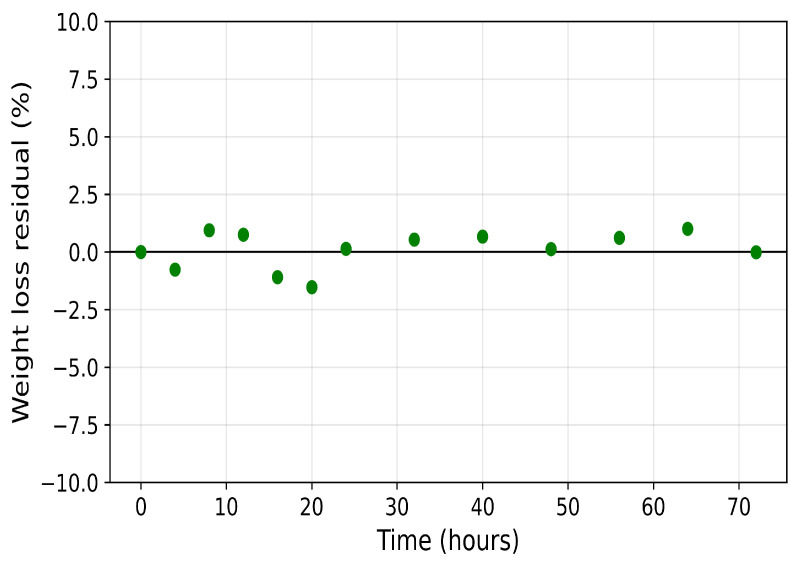
Residual plot for weight loss of PCL.

**Figure 6:**
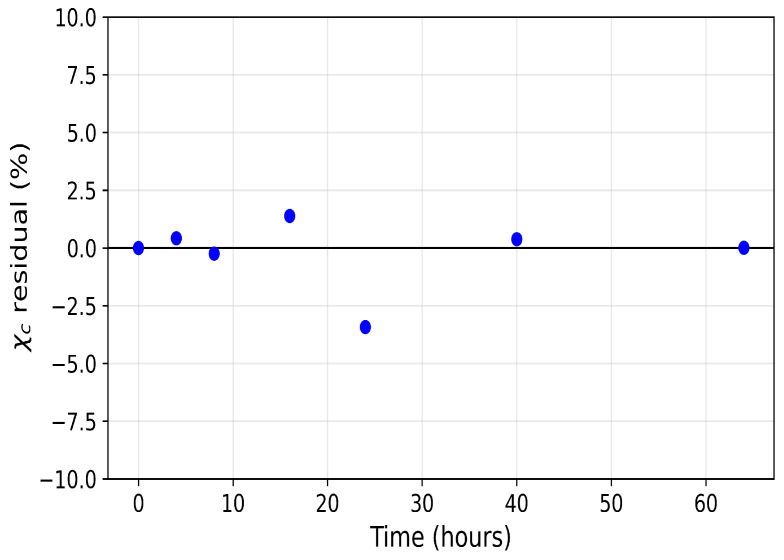
Residual plot for crystallinity of PCL.

Figure 5 and Figure 6 show the model residuals, defined at each observation time $t_{i}$ as $e_{i}=y_{i}\left(\theta \right)-y_{d,i}$, where $y_{i}\left(\theta \right)$ denotes the model prediction and $y_{d,i}$ the corresponding experimental measurement. For the weight-loss data (Figure 5), residuals are small and broadly centred near zero, with most falling within ±2%. The largest deviation is approximately −2% at t=20h. For the crystallinity data (Figure 6), the residual pattern is more informative. Residuals are near zero at early times (t=0, t=4,t=8h), increase to approximately +2% at t=16h, and exhibit a maximum magnitude of approximately −3.4% at t=24h. It is unclear why the observed crystalline content shows no statistically significant change between the 16 and 24 h time points before further crystallinity loss resumes at later stages. One possible explanation is that the remaining crystalline regions are more ordered and less accessible than those attacked initially, temporarily slowing the amorphisation process. Alternatively, degradation-induced recrystallisation of chain segments may partially offset ongoing crystallinity loss, as recrystallisation of short chains formed during polymer degradation is well documented in the literature [31,33,34].

Further studies are required to determine whether this behaviour is reproducible and to identify the underlying mechanisms. Regardless of the cause, the effect appears temporary, and the model yields small residuals across the full time domain. The smaller residuals at later times (t=40 and 64h) reflect the approach to a plateau and slower system dynamics, rather than a restriction of model applicability.

### 3.2. Parameter Uncertainty Analysis

**Figure 7:**
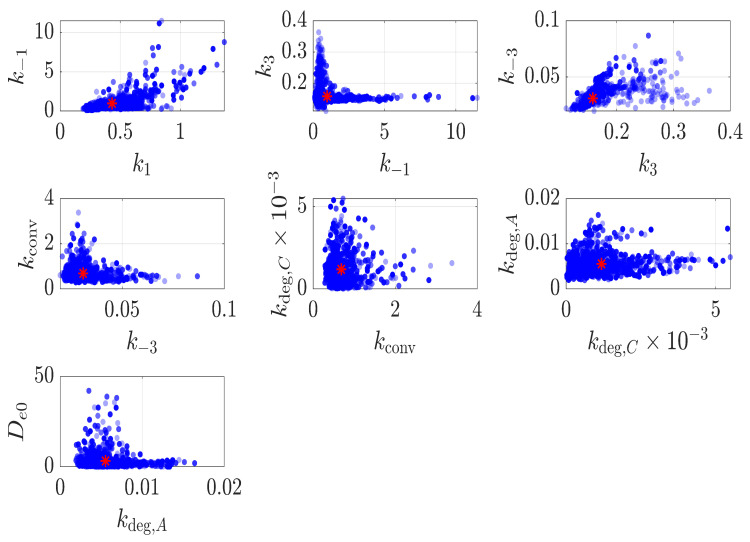
Pairwise scatter plots of posterior samples for all parameters. Red asterisks denote the posterior expected values of the parameter vector.

Bayesian parameter inference was used to quantify uncertainty in the estimated parameter vector θ. The prior distribution p(θ) was specified independently for each parameter as a log-uniform distribution over the feasible parameter domain D, using the same bounds (15) as in the least-squares fitting described in Section 3.1. This prior was combined with the likelihood function $p(y_{d}|\theta )$ to define the posterior distribution π(θ) according to Bayes’ theorem, as given in Equation (16). Standard deviations for the Gaussian measurement errors were taken from the experimental error bars reported by Shi et al. [16], and both weight-loss and crystallinity datasets were incorporated jointly in the likelihood, as described in Equation (17). MCMC sampling was applied using the Differential Evolution Markov Chain with snooker update (DE-MCz) algorithm [35], which employs 40 parallel chains initialised from the least-squares estimate. A total of 500 iterations were run with a thinning factor of 5, yielding 4000 posterior draws from which 95% credible intervals for each parameter were derived. Following the framework of [25,35], the posterior distribution is

(16) $$\pi \left(\theta \right)=p\left(\theta |y_{d}\right)=\frac{p\left(\theta \right)\;p\left(y_{d}|\theta \right)}{p(y_{d})},$$

where $p(y_{d}|\theta )$ denotes the likelihood of the observed data given parameters θ, and $p(y_{d})$ is the marginal likelihood that normalises the posterior. The joint likelihood over both observables is

(17) $$\begin{array}{cc} p(y_{d}|\theta )\; & =\prod_{i=1}^{n}\frac{1}{\sqrt{2\pi \sigma_{WL,i}^{2}}}\exp\;\left(-\frac{{\left({\hat{y}}_{WL,i}-y_{WL,i}\right)}^{2}}{2\sigma_{WL,i}^{2}}\right)\cdot \prod_{j=1}^{m}\frac{1}{\sqrt{2\pi \sigma_{\chi_{c},j}^{2}}}\exp\;\left(-\frac{{\left({\hat{y}}_{\chi_{c},j}-y_{\chi_{c},j}\right)}^{2}}{2\sigma_{\chi_{c},j}^{2}}\right), \end{array}$$

where ${\hat{y}}_{WL}\left(t_{i};\theta \right)$ and ${\hat{y}}_{\chi_{c}}\left(t_{j};\theta \right)$ are the model-predicted weight loss and crystallinity at times $t_{i}$ and $t_{j}$ respectively, obtained by numerical solution of Equations (2)–(7). The posterior parameter regions are shown in Figure 7 as pairwise plots ($\theta_{i+1}$ vs. $\theta_{i}$), with red asterisks marking the posterior means. These pairwise clouds reveal clear differences in how tightly each parameter is constrained by the Shi et al. [16] data.

**Figure 8:**
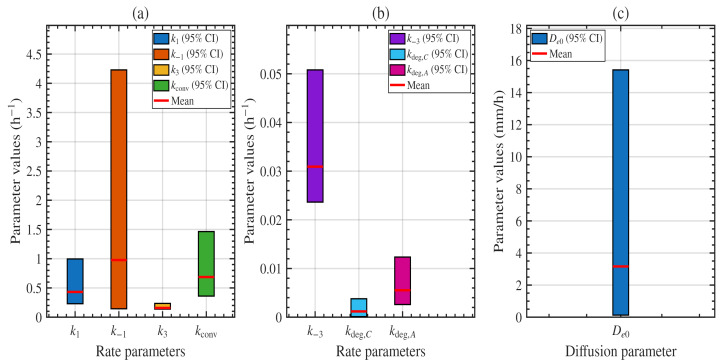
Posterior means with 95% credible intervals for the model parameters: (**a**) larger kinetic parameters $k_{1}$, $k_{-1}$, $k_{3}$, and $k_{\mathrm{conv}}$; (**b**) smaller kinetic parameters $k_{-3}$, $k_{\deg,C}$, and $k_{\deg,A}$; and (**c**) diffusion parameter $D_{e0}$. Parameters are shown on separate scales for visibility.

The marginal posterior uncertainty is summarised in Figure 8 through 95% credible intervals (CIs), while the posterior means are reported in Table 2. Among the rate parameters, the amorphous binding rate $k_{3}$, the crystalline degradation rate $k_{\deg,C}$, the amorphous degradation rate $k_{\deg,A}$, and the crystalline dissociation rate $k_{-3}$ exhibit the narrowest credible intervals and are therefore the most tightly constrained by the data. By contrast, the crystalline binding and dissociation rates, $k_{1}$ and $k_{-1}$, show substantially wider credible intervals, indicating greater uncertainty and no clear separation between them. For the amorphous pathway, however, the binding rate $k_{3}$ is more tightly constrained and larger than the reverse dissociation rate $k_{-3}$, suggesting more stable complex formation in the amorphous region. The posterior means also indicate that the amorphous degradation rate $k_{\deg,A}$ exceeds the crystalline degradation rate $k_{\deg,C}$, consistent with previous experimental studies showing faster degradation in amorphous domains [11,36]. In contrast, the diffusion coefficient $D_{e0}$ exhibits markedly greater uncertainty than any of the kinetic rate parameters. Overall, the credible interval analysis demonstrates that the dominant kinetic parameters are constrained to within approximately a single order of magnitude. The joint weight-loss and crystallinity dataset of Shi et al. [16] therefore provides sufficient information to constrain the main degradation processes and yield reliable model predictions. The broader uncertainty in $D_{e0}$ reflects the reaction-limited regime of the thin-film geometry used by Shi et al. [16]; this uncertainty might be reduced by conducting experiments at greater film thickness, where transport timescales become significant relative to reaction timescales.

### 3.3. Prediction Uncertainty

The impact of parameter uncertainty on predictive uncertainty was quantified using the framework of van Mourik et al. [35]. Posterior samples drawn as described in Section 3.2 were propagated through the model to generate an ensemble of trajectories, from which pointwise 95% credible bands were computed for the observables.

**Figure 9:**
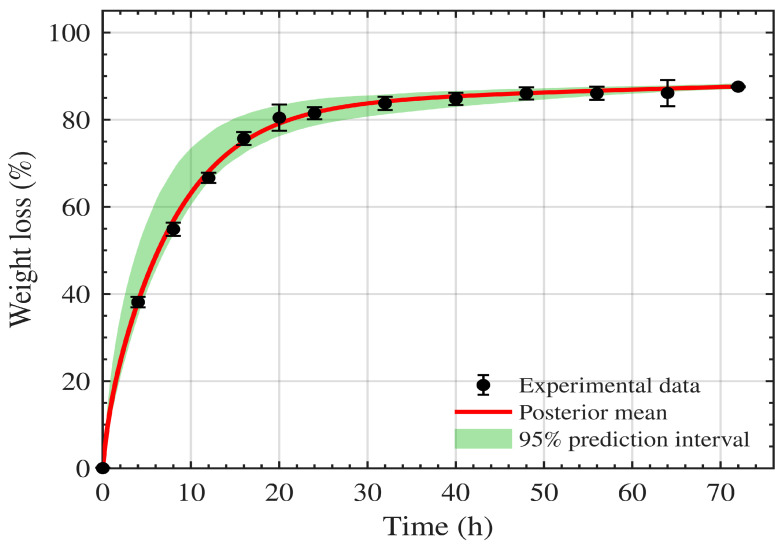
Model dynamics fitted to the Shi et al. [16] weight-loss data. The prediction uncertainty region is shown in green. The red curve is based on the expected parameters.

**Figure 10:**
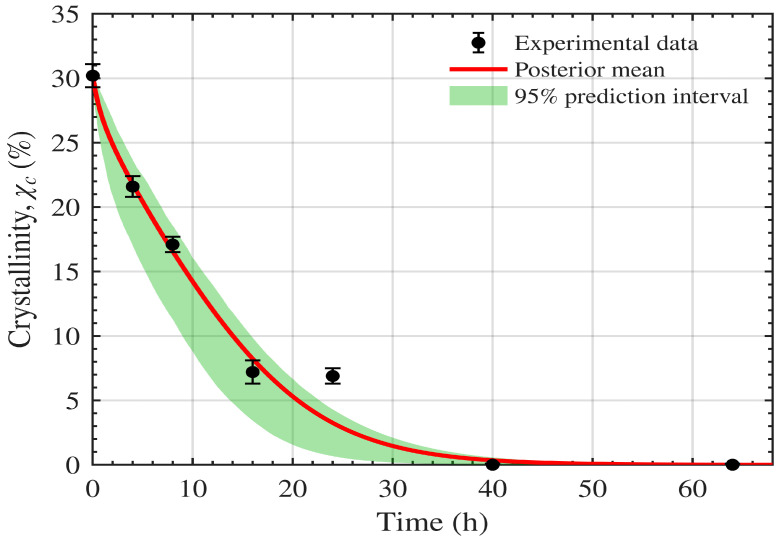
Model dynamics fitted to the Shi et al. [16] crystallinity data. The prediction uncertainty region is shown in green. The red curve is based on the expected parameters.

Figure 9 and Figure 10 show the resulting prediction uncertainty for weight loss and crystallinity $\chi_{c}\left(t\right)$ respectively, for the data of Shi et al. [16]. The red curves correspond to the posterior mean parameter vector, while the green shaded regions represent the 95% credible intervals of the predicted trajectories induced by posterior parameter uncertainty.

For weight loss (Figure 9), the credible band remains narrow over most of the time course and closely tracks the measurements, indicating that the parameter uncertainty produces only limited variability in the predicted macroscopic mass loss. For crystallinity (Figure 10), the model captures the overall decline, with modest uncertainty inflation at early times where the dynamics are steepest. However, the crystallinity loss at 24 h lies outside the uncertainty region, indicating that, as discussed in Section 5, the crystallinity dynamics are not fully captured by the current model formulation.

## 4. Global Sensitivity

To quantify the influence of model parameters on the predicted degradation responses, we performed a global sensitivity analysis using Sobol’s method. Sobol analysis is a variance-based technique that decomposes output variance into contributions from individual parameters and their interactions [37]. It therefore distinguishes first-order effects, which measure the independent contribution of each parameter, from total-order effects, which include both independent and interaction effects. Let $x=(x_{1},x_{2},\ldots ,x_{s})$ denote the model input parameters, each independently and uniformly distributed over [0,1]. The model output f(x) has mean $f_{0}$ and variance *D*, given by

(18) $$\begin{array}{c} f_{0}=\int f\left(x\right)\;dx,\;D=\int f{\left(x\right)}^{2}\;dx-f_{0}^{2}. \end{array}$$

Sobol’s method expresses f(x) as

(19) $$f\left(x\right)=f_{0}+\sum_{i=1}^{s}f_{i}\left(x_{i}\right)+\sum_{i=1}^{s}\sum_{\begin{array}{c} i\neq j \end{array}}^{s}f_{ij}\left(x_{i},x_{j}\right)+\cdots +f_{1,\ldots ,s}\left(x_{1},\ldots ,x_{s}\right),$$

which induces the variance decomposition

(20) $$D=\sum_{i=1}^{k}D_{i}+\sum_{i<j}D_{ij}+\sum_{i<j<l}D_{ijl}+\cdots +D_{1,2,\ldots ,k}.$$

Here, $D_{i_{1}\ldots i_{s}}$ is the partial variance associated with the parameter subset $\left(x_{i_{1}},\ldots ,x_{i_{s}}\right)$, and the corresponding Sobol sensitivity index is

(21) $$S_{i_{1}\ldots i_{s}}=\frac{D_{i_{1}\ldots i_{s}}}{D}.$$

In particular, the first-order index

(22) $$S_{i}=\frac{D_{i}}{D}$$

measures the independent contribution of parameter $x_{i}$, while the total-order index

(23) $$S_{T_{i}}=S_{i}+S_{ij,\;j\neq i}+\cdots +S_{1\ldots i\ldots s}$$

measures its overall contribution, including interactions. The sensitivity indices satisfy

(24) $$\sum_{i=1}^{k}S_{i}+\sum_{i<j}S_{ij}+\sum_{i<j<l}S_{ijl}+\cdots +S_{1,\ldots ,k}=1.$$

**Figure 11:**
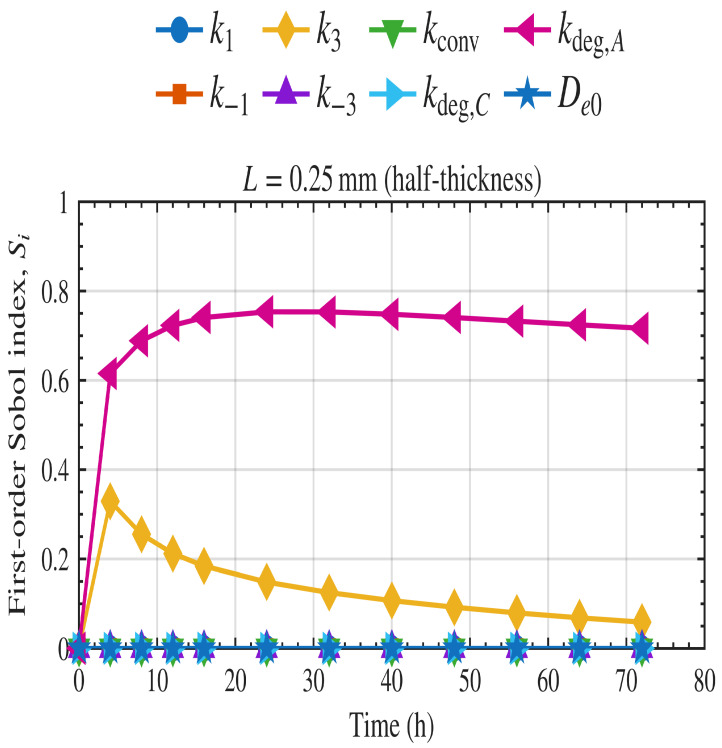
Time-dependent first-order Sobol sensitivity indices for film thickness of 0.5 mm.

**Figure 12:**
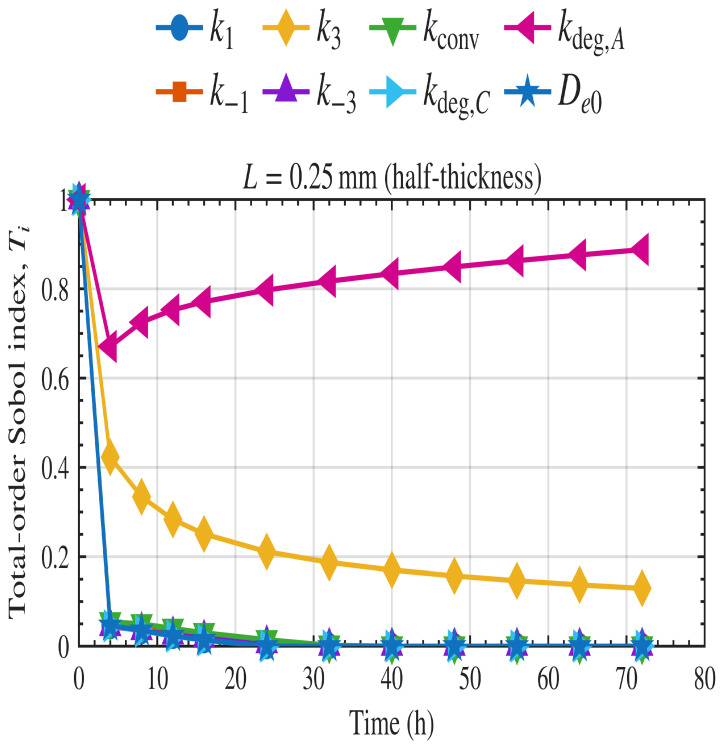
Time-dependent total-order Sobol sensitivity indices for film thickness of 0.5 mm.

Sobol analysis was implemented in MATLAB using the calibrated model in Equations (2)–(7) for the mass loss. Time-dependent first-order and total-order indices were computed for each kinetic parameter at the thickness of 0.5 mm used in the experimental study and are shown in Figure 11 and Figure 12, respectively.

In the first-order indices (Figure 11), $k_{\deg,A}$ is the dominant parameter throughout the simulation, indicating that when considering each parameter independently, the mass-loss rate is most sensitive to the rate of degradation of the amorphous region. The rate of enzyme binding to the amorphous region $k_{3}$ has a smaller but significant first-order contribution, particularly at earlier time points, before its influence gradually declines. By contrast, the remaining parameters, including $k_{-3}$, $k_{1}$, $k_{-1}$, $k_{\mathrm{conv}}$, $k_{\deg,C}$, and $D_{e0}$, show negligible first-order effects across the simulation window. A similar pattern is observed in the total-order indices (Figure 12). The parameter $k_{\deg,A}$ remains the dominant source of sensitivity even when interaction effects are included, while $k_{3}$ also retains a clear total-order contribution. The difference between the first-order and total-order roles of $k_{3}$ indicates that its influence is not purely independent, but also arises through interactions with other parameters. The other parameters contribute only minimally to total order, primarily at early time points, and become negligible as degradation progresses. Overall, these results indicate that the system is primarily controlled by the degradation of the amorphous regions.

### 4.1. Effect of Thickness on Parameter Sensitivity

**Figure 13:**
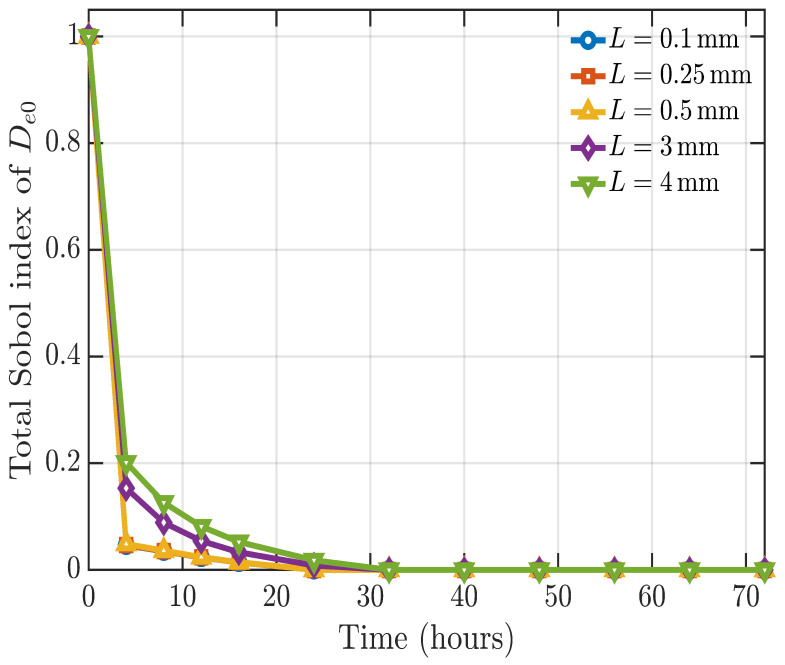
Total-order Sobol sensitivity indices for $D_{eo}$ for full film thicknesses of 0.2, 0.5, 1, 6, and 8mm. (Note *L* is half the thickness.)

The Sobol sensitivity analysis described above was repeated for different values of film thickness, both above and below the 0.5 mm thickness used in the experiment. The first-order Sobol sensitivity indices were found to be essentially independent of film thickness and follow the same pattern as shown in Figure 11. In contrast, the total-order sensitivity index of the diffusion parameter, $D_{e0}$, does vary with thickness (Figure 13). For the thinner films of L=0.5mm and below, the total order sensitivity index curves for the diffusion parameter overlap, indicating a similarly weak role of diffusion in this regime. For thicker films, the total-order sensitivity of $D_{e0}$ increases with thickness, showing that transport-related interaction effects become more important with increasing thickness. The total-order sensitivities of the other parameters are unaffected by thickness.

### 4.2. Effect of Film Thickness on Enzymatic Degradation

**Figure 14:**
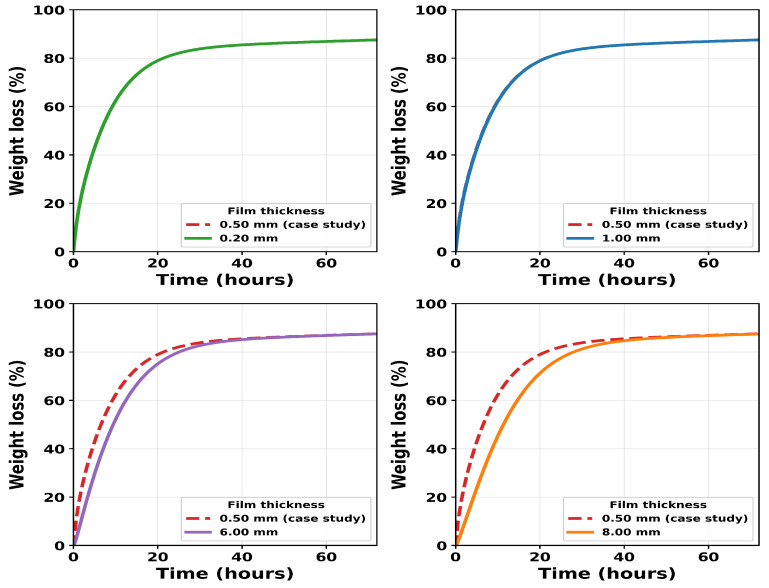
Predicted weight loss for film thicknesses of 0.20, 1.00, 6.00, and 8.00 mm in comparison to the weight loss of the 0.5 mm thickness film in the experimental case study of [16]. The predicted curves for the 0.20 mm and 1 mm films overlap that of the 0.5 mm film, indicating no change in the rate of % weight loss at these thickness values.

**Figure 15:**
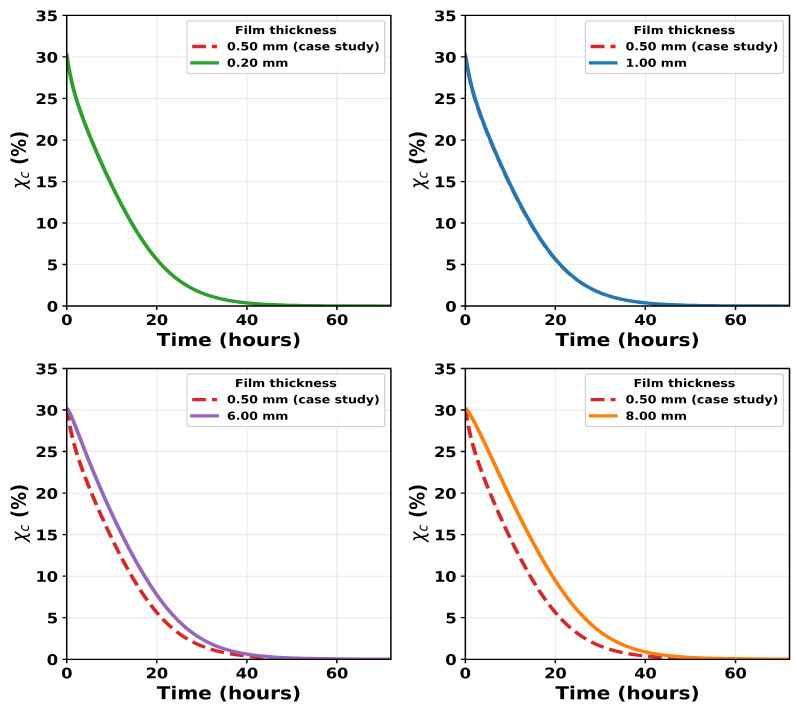
Predicted crystallinity ($\chi_{c}$) for film thicknesses of 0.20, 1.00, 6.00, and 8.00 mm in comparison to the crystallinity ($\chi_{c}$) of the 0.5 mm thickness film in the experimental case study of [16]. The predicted curves for the 0.20 mm and 1 mm films overlap that of the 0.5 mm film, indicating no change in the rate of % crystallinity ($\chi_{c}$) at these thickness values.

We use the identified model to explore the effect of film thickness on the rates of mass and crystallinity loss. All calibrated parameters in Table 1 were fixed and only the film thickness was varied. The model is solved on a symmetric half-domain, x∈[0,L], where *L* is the half-thickness and the full physical film thickness is 2L. Figure 14 and Figure 15 show results for L=0.10, 0.25, 0.50, 3.00, and 4.00 mm, corresponding to full thicknesses of 0.20, 0.50, 1.00, 6.00, and 8.00 mm, respectively. The experimental case study of Shi et al. [16] corresponds to a full thickness of 0.50 mm in the model.

To compare reaction and transport, we define Damköhler numbers for the amorphous and crystalline domains:

(25) $$Da_{A}=\frac{k_{\deg,A}L^{2}}{D_{e0}},\;Da_{C}=\frac{k_{\deg,C}L^{2}}{D_{e0}}.$$

The Damköhler number is a dimensionless ratio of the catalytic timescale to the diffusion timescale. Since $k_{\deg,A}>k_{\deg,C}$, the amorphous pathway has the shorter characteristic catalytic timescale and therefore governs the onset of diffusion-limited behaviour. The thickness dependence is thus primarily controlled by $Da_{A}$.

For the thinner films (0.20, 0.50, and 1.00 mm), the predicted weight-loss and crystallinity curves are nearly identical to those of the case study. This indicates reaction-limited behaviour, where enzyme diffusion across the film is much faster than catalytic degradation and the enzyme concentration remains approximately uniform throughout the film. In this regime, the diffusion timescale $\tau_{\mathrm{diff}}=L^{2}/D_{e0}$ is small (approximately 0.006–0.04 h). It should be noted that the uncertainty in $D_{e0}$ is relatively high (Figure 8c), so these values should be interpreted as indicative rather than precise.

As thickness increases, the predictions deviate progressively from the case-study curve in both mass loss and crystallinity. Weight loss slows and crystallinity declines more gradually because the enzyme must diffuse further before degradation and amorphisation can proceed in the film interior. At L=4.00 mm (full thickness 8.00 mm), $\tau_{\mathrm{diff}}\approx 9.7$ h, so diffusion is no longer negligible relative to the catalytic timescale and $Da_{A}$ approaches unity. This marks the transition to transport-influenced behaviour.

Below a critical thickness, mass loss and crystallinity evolution are insensitive to film geometry because enzyme diffusion across the film is rapid relative to the catalytic timescale and the film is effectively enzyme-saturated. Above this thickness, further increases in *L* progressively slow both mass loss and crystallinity evolution because the time required for the enzyme to diffuse into the bulk becomes significant relative to the reaction timescale. To our knowledge, this geometry-dependent transition has not been directly demonstrated experimentally for the enzymatic degradation of PCL, and the present model predictions therefore provide motivation for multi-thickness degradation experiments to validate this behaviour.

**Figure 16:**
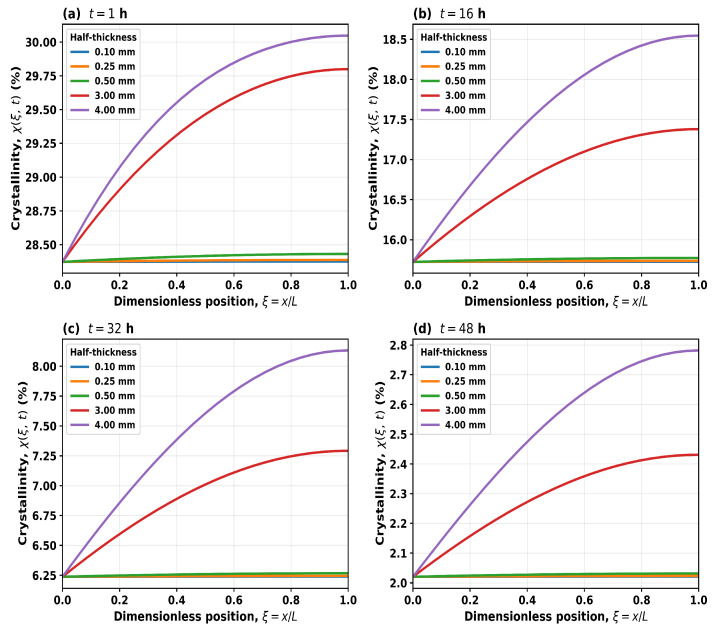
Spatial profiles of local crystallinity χ(ξ,t) (%) versus dimensionless position ξ=x/L, where 0 is the surface, and 1 is the centre of the film.

In addition to the bulk crystallinity χ(t), the local crystallinity is defined as

$$\chi \left(x,t\right)=\frac{C(x,t)}{C(x,t)+A(x,t)},$$

which represents the crystalline fraction of the remaining polymer at position *x* and time *t*. To further examine the internal evolution of the polymer microstructure, the spatial distribution of crystallinity χ(x,t) was evaluated across the film thickness (Figure 16). For thin films (L≤0.5 mm), the crystallinity profiles remain nearly uniform across the domain at all times, indicating that enzyme diffusion is sufficiently rapid to maintain an approximately homogeneous enzyme concentration, consistent with a reaction-limited regime.

In contrast, thicker films (L≥3 mm) exhibit spatial gradients in χ(x,t), with higher crystallinity retained in the interior (x≈L) compared to the surface (x=0). This behaviour reflects a lower enzyme concentration in the core relative to the surface at early stages of degradation, rather than a complete absence of enzyme in the bulk. As degradation progresses, these differences diminish, leading to more uniform profiles at later times. The magnitude of the spatial gradients increases with film thickness, consistent with increased diffusion timescales and corresponding changes in $D_{A}$. These spatial profiles therefore provide mechanistic insight into the transition from reaction-limited to transport-influenced degradation, as also reflected in the global metrics presented in Figure 14 and Figure 15.

## 5. Discussion

This study addresses a key gap in enzymatic degradation modelling of semicrystalline polymers. Existing enzymatic models can reproduce mass-loss trends, but they usually treat the polymer as a single homogeneous state. As a result, they cannot resolve state-selective degradation, evolution of the crystalline fraction over time, or the coupling between transport and microstructural change. The objective of the present work was to overcome this limitation by developing a reaction–diffusion model in which crystalline and amorphous regions are represented separately, and enzyme mobility depends on porosity which increases as degradation proceed. The results show that this framework can reproduce both mass loss and evolution of the crystalline fraction and can therefore address mechanistic questions that cannot be resolved from mass-loss data alone. In semicrystalline polymers, the same mass-loss profile may arise from different underlying mechanisms, including preferential degradation of the amorphous state, more balanced attack on both states, or disruption of crystalline domains before complete hydrolysis. By incorporating crystallinity as a second response, the model distinguishes between these possibilities and provides a more informative description of the degradation mechanism. The amorphisation rate estimated from the data supports the interpretation that *Candida antarctica* lipase does not act only on the amorphous fraction.

The model also provides a mechanistic explanation for the experimentally observed degradation profile, supported by good agreement with both the weight-loss and crystallinity data. The main discrepancy is localised around 24 h, where the model does not reproduce the short plateau in the crystalline fraction seen in the experimental data. This plateau, which is absent from the weight-loss data, may be due to heterogeneity in the accessibility of the crystalline regions, with those with more loosely packed lamellae being disrupted more quickly by the enzyme. Alternatively, as degradation produces highly mobile short-chain oligmers, these may form new crystalline regions which temporarily offset the amorphisation process.

The posterior summaries indicate that the kinetic parameters are better constrained by the data than the diffusion parameter. This is consistent with the case-study geometry, since the thin films used by Shi et al. [16] operate in a regime where enzyme transport is rapid relative to catalytic degradation. Under such conditions, the mass loss and crystalline fraction measurements carry little information about the transport timescale. This has a clear implication for designing future experiments with the aim of accurate identification of both reaction and diffusion parameters. In such cases, it would be beneficial to conduct the degradation experiment on a thicker film, which will ensure the timescale for enzyme diffusion is significant relative to the timescale for the degradation reaction. The sensitivity analysis reveals how the relative importance of kinetic mechanisms shifts across the degradation timeline. Catalytic degradation of the amorphous state is the dominant main effect at early stages, while enzyme binding becomes increasingly significant at later stages through its interaction with the catalytic step. These results provide mechanistic insight into which mechanisms govern each stage of degradation.

The thickness study identifies a transition between reaction-limited and transport-influenced degradation. Below a certain threshold thickness, the predicted weight-loss and crystallinity curves are almost unchanged as thickness varies, showing that enzyme diffusion is fast relative to reaction and that geometry has little influence on the overall degradation timescale. For thicknesses above this threshold, enzyme penetration into the interior becomes limiting, and the model predicts slower mass loss and a more gradual decline in crystallinity as a result. Since the design thickness of a bioresorbable film would be determined by mechanical requirements, these predictions are relevant to anticipating how a given geometry will behave in service. This includes both the rate of mass loss and the evolution of the crystalline fraction, which together govern how the mechanical properties of the device change over time during resorption.

The one-dimensional formulation is appropriate for the thin-film geometry studied here, where through-thickness gradients dominate, and lateral variations at the edges can be neglected. In thicker or more compact geometries, or whenever edge effects and lateral transport are significant, a two-dimensional or three-dimensional model would be needed. The linear porosity–diffusivity relation is also a simplifying approximation of how the porosity of the film evolves during degradation. While the assumption that the soluble products are cleared instantaneously is justified for thin film, and well-mixed systems of [16], this may not hold in thicker geometries or in vivo, where local product accumulation could engage product-inhibition kinetics [25,28,38]. The optimal degradation condition depends on the specific application and is governed by a combination of material properties and enzymatic conditions. In particular, geometry influences transport: thinner films tend to allow rapid enzyme penetration and may exhibit bulk degradation, whereas thicker materials are more likely to show more surface-localised behaviour. However, the degradation regime also depends on the enzyme mechanism, as different enzymes (e.g., lipases versus cutinases) can induce markedly different erosion behaviours even for the same material. In addition, material properties such as molecular weight can influence degradation behaviour and may affect the identified kinetic and transport parameters. The present model is calibrated to a specific PCL material and experimental dataset, and the fitted parameters should therefore be interpreted as effective parameters for this system. Extension of the framework to different materials or molecular weights would likely require not only recalibration but also potential reformulation of the model structure to reflect differences in degradation mechanisms. For example, this may include removing the amorphisation step or incorporating additional processes such as recrystallisation, depending on the material behaviour. The Shi et al. [16] study used a single thin-film geometry, in which enzyme diffusion is fast relative to reaction. Therefore, the data carry little information on the diffusion term, and hence its broader posterior (Section 3.2). With no thicker-film data available in [16], the role of thickness was instead explored predictively using the identified parameters (Section 4.2).

Extension of the model to in vivo settings, where local pH and temperature may vary within physiologically relevant ranges and could modulate enzyme activity, would require explicit coupling of these environmental variables to the reaction kinetics and enzyme transport, and is a natural direction for future work.

## 6. Conclusions

We present a mechanistic reaction–diffusion model for enzymatic degradation of a semicrystalline polymer in which crystalline and amorphous states are treated separately, and crystallinity evolves dynamically. Calibrated to in vitro PCL–*Candida antarctica* lipase data, the model reproduced both the weight-loss profile and the overall decline in crystallinity. Uncertainty analysis showed that the thin-film dataset constrained the reaction-rate parameters much more strongly than the diffusion parameter, consistent with a largely reaction-limited regime. The model allows for exploration of how film thickness affects the rate of mass loss and microstructure evolution: thin films remain largely reaction-limited, whereas thicker specimens become transport-influenced, with slower degradation and longer retention of a crystalline interior. These findings are important for implant and device design because degradation rate, evolution of the crystalline fraction, and enzyme access together influence structural integrity, resorption timescale, and release behaviour (for drug eluting devices).

## Figures and Tables

**Table 1:** Estimated parameters for the model.

$k_{1}\;\left(h^{-1}\right)$	$k_{-1}\;\left(h^{-1}\right)$	$k_{3}\;\left(h^{-1}\right)$	$k_{-3}\;\left(h^{-1}\right)$	$k_{\mathrm{conv}}\;\left(h^{-1}\right)$	$k_{\deg,C}\;\left(h^{-1}\right)$	$k_{\deg,A}\;\left(h^{-1}\right)$	$D_{e0}\;\left(\mathrm{mm}/h\right)$
0.65	0.47	0.30	0.03	0.58	0.0012	0.005	1.66

**Table 2:** Posterior mean parameter estimates from Bayesian inference.

$k_{1}\;\left(h^{-1}\right)$	$k_{-1}\;\left(h^{-1}\right)$	$k_{3}\;\left(h^{-1}\right)$	$k_{-3}\;\left(h^{-1}\right)$	$k_{\mathrm{conv}}\;\left(h^{-1}\right)$	$k_{\deg,C}\;\left(h^{-1}\right)$	$k_{\deg,A}\;\left(h^{-1}\right)$	$D_{e0}\;\left(\mathrm{mm}\;h^{-1}\right)$
0.43	0.98	0.16	0.031	0.69	0.0012	0.006	3.16

## Data Availability

The code supporting the findings of this study and the resulting data generated are openly available on GitHub at https://github.com/nansaknanshin-collab/pcl-degradation-model and archived on Zenodo (DOI: 10.5281/zenodo.19347579).
